# Eleven-Year Trends in Antimicrobial Resistance and Outcomes Among Hospitalized Adults with Positive *Enterococcus* Cultures

**DOI:** 10.3390/microorganisms14102175

**Published:** 2026-09-27

**Authors:** Esraa Aldawood, Hamad M. Almatrudi

**Affiliations:** Department of Clinical Laboratories Sciences, The College of Applied Medical Sciences, King Saud University, Riyadh 12372, Saudi Arabia; halmatrudi1@ksu.edu.sa

**Keywords:** vancomycin-resistant enterococci, *Enterococcus faecium*, *Enterococcus faecalis*, antimicrobial resistance, healthcare-associated infections, mortality

## Abstract

Background: Enterococcal infections are major healthcare-associated infections associated with increasing antimicrobial resistance and substantial mortality. However, long-term epidemiological data integrating antimicrobial resistance trends with clinical outcomes remain limited in Saudi Arabia. This study evaluated antimicrobial resistance trends, clinical characteristics, and mortality among patients with enterococcal positive cultures over an 11-year period. Methods: We conducted a single-center retrospective cohort study of 2272 hospitalized adults with culture-positive *Enterococcus* spp. between March 2015 and November 2025. Demographic, clinical, microbiological, and outcome data were extracted from electronic medical records. Temporal resistance trends and independent predictors of in-hospital mortality were evaluated using logistic regression. Results: *Enterococcus faecalis* accounted for 74.1% of isolates. Vancomycin-resistant enterococci (VRE) prevalence showed no significant temporal trend during the study period, with an overall prevalence of 11.3%, whereas high-level gentamicin resistance declined significantly (OR per year 0.933, 95% CI 0.903–0.964; *p* < 0.001). Multidrug resistance showed no significant temporal change. Compared with *E. faecalis*, *E. faecium* exhibited substantially higher resistance to vancomycin, ampicillin, and ciprofloxacin, while susceptibility to linezolid, daptomycin, and tigecycline remained above 95%. Overall, in-hospital mortality was 15.6% and was independently associated with age ≥60 years, intensive care unit admission, bloodstream infection, coinfection, and *E. faecium* positive culture. Conclusions: This study provides one of the largest long-term evaluations of enterococcal positive culture in Saudi Arabia, demonstrating stable VRE prevalence, declining high-level aminoglycoside resistance, and persistent species-specific differences in antimicrobial resistance and mortality. These findings support local antimicrobial resistance surveillance and antimicrobial stewardship efforts by providing updated epidemiological data on enterococcal positive cultures.

## 1. Introduction

Enterococci are commensal organisms of the gastrointestinal tract but have emerged as important causes of healthcare-associated infections, including urinary tract infections, wound infections, intra-abdominal infections, and device-associated infections, which may progress to severe conditions such as bacteremia and endocarditis [1]. The most common clinical species that cause significant infections are *Enterococcus faecalis* and *Enterococcus faecium* [2,3,4,5].

The emergence and spread of multidrug-resistant pathogens represent a major global health challenge, significantly limiting therapeutic options and increasing morbidity and mortality worldwide [6]. In this context, enterococci are particularly concerning due to their intrinsic resistance to multiple antimicrobial classes, including cephalosporins, semisynthetic penicillinase-stable penicillins, clindamycin, and low-level aminoglycosides, as well as their ability to acquire additional resistance determinants. Unlike many other Gram-positive bacteria, enterococci exhibit intrinsic tolerance to several cell wall-active antibiotics, meaning that agents which are typically bactericidal against other Gram-positive organisms often demonstrate only bacteriostatic activity against enterococci, necessitating combination therapy for serious infections such as infective endocarditis [7,8]. The emergence of vancomycin-resistant enterococci (VRE) has further compounded these challenges and remains a major concern in healthcare settings. For VRE infections, ampicillin may still be used when isolates remain susceptible. In the United States, vancomycin-resistant *Enterococcus faecium* isolates are frequently associated with ampicillin resistance, whereas *Enterococcus faecalis* isolates are more commonly susceptible to ampicillin [9,10,11]. In Saudi Arabia, resistance patterns vary across healthcare institutions; however, genomic data from a Saudi tertiary-care hospital suggest that vancomycin-resistant *Enterococcus faecium* isolates commonly exhibit resistance to ampicillin, whereas *Enterococcus faecalis* isolates are more frequently susceptible to ampicillin [12]. The emergence of ampicillin-resistant VRE further restricts treatment options, leaving linezolid and daptomycin as the main therapeutic agents, while quinupristin/dalfopristin and tigecycline may be considered in selected clinical scenarios [12].

The COVID-19 pandemic was associated with widespread antibiotic use despite the relatively low prevalence of bacterial coinfections, raising concerns regarding its potential impact on antimicrobial resistance. Several studies have highlighted increased empirical antibiotic prescribing and antimicrobial misuse during the pandemic, emphasizing the importance of continued antimicrobial stewardship and surveillance in the post-pandemic era [13,14]. Despite this, data on temporal changes in enterococcal resistance patterns in Saudi Arabia remain limited. A previous study from the southern region of Saudi Arabia reported a VRE prevalence of 15.9% over a 10-year period [15], while data from Riyadh are scarce and largely exist before the COVID-19 pandemic [16].

Therefore, this study aimed to (i) evaluate the demographic and clinical characteristics of adult patients with enterococcal positive culture before and during/after the onset of the COVID-19 pandemic, (ii) identify factors associated with in-hospital mortality, and (iii) assess long-term trends in antimicrobial resistance among *Enterococcus* spp. over an 11-year period in a tertiary care hospital in Riyadh, Saudi Arabia.

## 2. Materials and Methods

### 2.1. Study Design

This retrospective observational study was carried out at King Khalid University Hospital, a major teaching and research center within the King Saud University Medical City (KSUMC) in Riyadh, Saudi Arabia, which provides comprehensive healthcare services, including specialized oncology and surgical care. Adult hospitalized patients (≥18 years) with at least one positive clinical culture for *Enterococcus* spp. between March 2015 and November 2025 were identified from the microbiology laboratory database. For temporal analysis, the study period was categorized into a pre-pandemic period (March 2015–February 2020) and a pandemic/post-pandemic period (March 2020–November 2025). March 2020 was selected as the transition point because it corresponds to the onset of the COVID-19 pandemic in Saudi Arabia and the implementation of major public health interventions and healthcare system adaptations. This classification was used to explore temporal differences in Enterococcus epidemiology and antimicrobial resistance patterns and was not intended to establish a causal association between the COVID-19 pandemic and observed changes. All positive clinical Enterococcus cultures obtained during routine patient care were eligible for inclusion. Collected variables included demographic data (age and sex), clinical characteristics (underlying conditions and comorbidities), ward type, presence and type of urinary catheter, site of isolation, and microbiological data, including species identification and antimicrobial susceptibility profiles. In-hospital mortality was defined as death occurring during the same hospitalization in which an Enterococcus-positive culture was isolated. Thirty-day mortality was defined as death occurring within 30 days of the index Enterococcus-positive culture based on the KKUH electronic medical record system. All patient data were handled confidentially and anonymized prior to analysis.

### 2.2. Study Population

Adult patients with laboratory-confirmed Enterococcus-positive cultures identified during the study period were eligible for inclusion. The study population comprised hospitalized patients from both ICU and non-ICU settings, with only the first isolate per patient included in the analysis.

Patients were excluded if duplicate isolates from the same individual were identified, if clinical data were incomplete, or if the isolates were obtained from outpatient sources.

### 2.3. Data Curation

Clinical and demographic data were obtained from electronic medical records. Demographic variables included age and sex. Clinical variables comprised admission ward (ICU or non-ICU), presence of comorbidities, presence and type of urinary catheter, source of isolation, admission and discharge dates, and clinical outcomes.

Microbiological data included the type of clinical specimen, presence of co-infection, date of Enterococcus isolation, and antimicrobial susceptibility results. Coinfection was defined as the isolation of at least one additional microorganism from any clinical specimen within 24 h of the index *Enterococcus*-positive culture. Owing to the retrospective study design and the lack of complete clinical information, this definition was based on microbiological findings and could not distinguish true polymicrobial infection from colonization or contamination in every case.

### 2.4. Microbiological Analysis and Antimicrobial Susceptibility Testing

Bacterial identification and antimicrobial susceptibility testing were performed using the MicroScan WalkAway Plus System with the Pos Breakpoint Combo Panel Type 28 (Beckman Coulter, Brea, CA, USA), according to the manufacturer’s instructions. Clinical microbiology specimens were processed and reported according to the hospital microbiology laboratory’s standard operating procedures. Reporting of organisms was specimen-specific and based on established laboratory criteria, including specimen quality, colony count (where applicable), mixed microbial growth, and the clinical significance of isolated organisms. Interpretation of antimicrobial susceptibility results (susceptible, intermediate, or resistant) was performed in accordance with the Clinical and Laboratory Standards Institute (CLSI) guidelines (latest available edition during the study period). The panel of antibiotics tested was selected based on their clinical relevance in the treatment of Enterococcus infections and their inclusion in both international and local empirical therapy guidelines. For categorical antimicrobial susceptibility analyses, the denominator for each antimicrobial agent was the number of Enterococcus isolates recovered from hospitalized adult patients with an interpretable susceptibility result (susceptible, intermediate, or resistant). Missing and unclassified results were excluded from antimicrobial-specific percentage calculations. MDR was defined according to the international consensus definition as acquired non-susceptibility to at least one antimicrobial agent in three or more antimicrobial categories [17]. High-level aminoglycoside resistance (HLAR) was defined as resistance to high concentrations of aminoglycosides, including high-level gentamicin resistance and/or high-level streptomycin resistance. When analyzed separately, high-level gentamicin resistance (HLGR) and high-level streptomycin resistance (HLSR) were reported individually.

### 2.5. Statistical Analysis

Statistical analyses were performed using GraphPad Prism version 10.6.1 (GraphPad Software, San Diego, CA, USA). Categorical variables were summarized as frequencies and percentages, whereas continuous variables were expressed as median and interquartile range (IQR).

Comparisons between groups were conducted using the chi-square test or Fisher’s exact test for categorical variables, as appropriate, and the Mann–Whitney U test for continuous variables and *p*-values < 0.05 were considered statistically significant.

Multivariable logistic regression analysis was performed to evaluate factors associated with in-hospital mortality. Variables were selected a priori based on their clinical relevance and previous evidence demonstrating their association with outcomes in patients with enterococcal infections [18,19]. These variables included age group, ICU admission, urinary catheter status, site of isolation, study period (pre-pandemic versus pandemic/post-pandemic), coinfection, and *Enterococcus* species. Results are reported as adjusted odds ratios (AORs) with 95% confidence intervals (CIs).

Temporal trends in antimicrobial resistance were assessed using logistic regression models with calendar year treated as a continuous variable. Results were presented as observed annual proportions alongside model-derived trend estimates.

### 2.6. Ethical Approval

Ethical approval was obtained from the Institutional Review Board (IRB) of KKU Hospital, IRB No. E-24-9024. Patients’ confidentiality was maintained, and no personal identifiers were used.

## 3. Results

### 3.1. Patient Characteristics of Enterococcal Positive Culture Pre-Pandemic and Pandemic/Post-Pandemic Period

A total of 2272 adult patients with enterococcal positive culture were included, with a median age of 59 years and a majority of females (56.7%) (Table 1). *Enterococcus faecalis* was the most frequently isolated species (74.1%), followed by *Enterococcus faecium* (20.9%). Overall, VRE was identified in 256 of 2261 isolates (11.3%), HLAR in 40.4%, and multidrug resistance (MDR) in 33.1%. The median length of hospital stay was 16.9 days, with in-hospital and 30-day mortality rates of 15.6% and 7.2%, respectively.

Compared to the pre-pandemic period, the pandemic/post-pandemic period showed a higher proportion of male patients (45.7% vs. 40.5%, *p* = 0.013), with no significant differences in age distribution. Significant shifts in clinical setting were observed, including increased ICU admissions (5.4% vs. 1.6%, *p* < 0.001) and medical ward cases (8.4% vs. 1.5%, *p* < 0.001), alongside a marked reduction in emergency department presentations (31.5% vs. 63.8%, *p* < 0.001).

The presence of urinary catheters decreased significantly during the pandemic/post-pandemic period (3.3% vs. 11.1%, *p* < 0.001), while catheter type distribution remained unchanged overall. Among comorbidities, chronic kidney disease increased (0.8% vs. 0.1%, *p* = 0.023), whereas end-stage renal disease decreased (0.8% vs. 2.1%, *p* = 0.010); other comorbidities were similar between groups.

No significant differences were observed in species distribution or VRE rates. However, HLAR decreased significantly in the pandemic/post-pandemic period (36.7% vs. 44.4%, *p* < 0.001), while MDR prevalence did not change. Clinical outcomes, including length of stay and mortality, did not differ significantly. In contrast, coinfections were significantly less frequent in the pandemic/post-pandemic period (0.9% vs. 8.8%, *p* < 0.001), particularly with *Klebsiella pneumoniae* and *Candida albican*.

### 3.2. Factors Associated with In-Hospital Mortality in Patients with Enterococcal Positive Culture

Multivariable logistic regression analysis identified several factors significantly associated with in-hospital mortality among patients with enterococcal positive culture (Table 2). Increasing age was strongly associated with mortality, with patients aged 40–59 years having 2.77-fold higher odds of death compared with those aged 18–39 years (AOR = 2.77, 95% CI: 1.60–4.98; *p* = 0.0002), while patients aged ≥60 years had a more than fivefold increased risk of mortality (AOR = 5.06, 95% CI: 3.08–8.72; *p* < 0.0001).

ICU admission was the strongest predictor of mortality. Patients admitted to the ICU had significantly higher odds of death compared with non-ICU patients (AOR = 28.1, 95% CI: 19.5–41.1; *p* < 0.0001).

Regarding the site of isolation, bloodstream positive cultures were significantly associated with increased mortality compared with urinary tract (AOR = 2.69, 95% CI: 1.84–3.92; *p* < 0.0001). In contrast, wound/soft tissue isolation, respiratory isolation, and other isolation sites were not significantly associated with mortality.

The Pandemic/post-pandemic period was not significantly associated with mortality compared with the pre-pandemic period (AOR = 1.18, 95% CI: 0.87–1.61; *p* = 0.26). Similarly, the absence of a urinary catheter was not significantly associated with mortality (AOR = 1.06, 95% CI: 0.60–1.90).

Patients without coinfection had significantly lower odds of mortality compared with those with coinfection (AOR = 0.50, 95% CI: 0.27–0.97; *p* = 0.041). In terms of *Enterococcus* species, infections caused by *Enterococcus faecalis* were associated with significantly lower mortality compared with *Enterococcus faecium*-positive culture (AOR = 0.51, 95% CI: 0.37–0.70; *p* < 0.0001), whereas other *Enterococcus* species were not significantly associated with mortality.

### 3.3. Antimicrobial Resistance Among Enterococcus spp. (Between March 2015 and November 2025)

Across the 11-year study period, *Enterococcus* spp. demonstrated variable antimicrobial resistance patterns, with an overall profile characterized by high susceptibility to linezolid (98.1%), daptomycin (95.9%), and tigecycline (99.6%), and moderate susceptibility to vancomycin (88.4%) and ampicillin (78.5%) (Supplementary Table S1), which also reports the numbers of isolates tested and resistant for each antimicrobial. In contrast, high resistance rates were observed for erythromycin (69.6%) and tetracycline (66.0%), while fluoroquinolones showed intermediate resistance levels (ciprofloxacin 35.6%, levofloxacin 33.2%). High-level gentamicin and streptomycin (synergy) resistance were detected in 27.4% and 29.1% of isolates tested for those agents, respectively. Four *E. faecium* isolates exhibited concurrent resistance to vancomycin and linezolid.

Temporal analysis (Figure 1 and Figure 2) showed that resistance patterns for ampicillin, ciprofloxacin, and vancomycin remained largely stable over time, with only minor fluctuations across years. In contrast, high-level gentamicin resistance (HLG) exhibited a consistent decline throughout the study period, as visualized in both the heat map and line graph.

These observations were confirmed by regression analysis (Table 3), which demonstrated a significant annual decrease in HLG resistance (OR 0.933 per year, 95% CI 0.903–0.964, *p* < 0.001). No significant temporal trends were identified for ampicillin (OR 1.026, *p* = 0.149), ciprofloxacin (OR 1.000, *p* = 0.993), or vancomycin (OR 1.001, *p* = 0.971), indicating stable resistance patterns over the 11-year period.

Resistance rates for ampicillin, ciprofloxacin, HLG, and vancomycin were stratified by species. A marked difference in antimicrobial resistance profiles was observed between *Enterococcus faecalis* and *Enterococcus faecium*. *E. faecium* demonstrated substantially higher resistance rates compared with *E. faecalis* to most tested antibiotics (Figure 3). Ampicillin resistance was significantly higher in *E. faecium* (82.0%) compared to *E. faecalis* (3.6%) (*p* < 0.0001). A similar pattern was observed for vancomycin, where resistance reached 42.9% in *E. faecium* versus 1.6% in *E. faecalis* (*p* < 0.0001). Ciprofloxacin resistance was also markedly higher in *E. faecium* (76.4%) compared with *E. faecalis* (24.8%) (*p* < 0.0001).

For HLGR, resistance was moderate in both species but remained significantly higher in *E. faecium* (32.0%) than in *E. faecalis* (27.1%) (*p* = 0.0462). Overall, *E. faecium* consistently exhibited a more multidrug-resistant phenotype compared with *E. faecalis*, particularly for ampicillin, vancomycin, and ciprofloxacin.

## 4. Discussion

In the present study, we retrospectively investigated the prevalence of enterococcal positive culture among hospitalized patients in a tertiary care hospital in Riyadh during the pre-pandemic and the pandemic/post-pandemic-eras. The prevalence of VRE in our cohort was 11.3%, which is lower than the 17.3% reported in a previous one-year study conducted at the same hospital during 2014–2015 [14]. It is also lower than the 15.9% VRE prevalence reported in a single-center study conducted in the southern region of Saudi Arabia between 2012 and 2021 [15]. In contrast, it is nearly double the prevalence reported from a tertiary hospital in the eastern region (6.7%) [20]. Our findings are comparable to those reported from Qatar, where a VRE prevalence of 10.6% was observed [21].

Furthermore, the VRE prevalence observed in our study falls within the lower range of rates reported in Europe, where substantial geographic variability has been documented. A meta-analysis reported VRE prevalence rates ranging from approximately 11% in Germany to nearly 40% in Turkey [22]. In comparison, VRE rates in the United States are generally higher, frequently exceeding 20% [23], whereas substantially lower rates, often below 2%, have been reported in China [24].

The COVID-19 pandemic was associated with an increase in ICU admissions among patients with enterococcal positive cultures, accompanied by a reduction in cases presenting through the emergency department. The most isolated *Enterococcus* spp. in pre-pandemic and in the pandemic/post-pandemic periods was *Enterococcus faecalis*, accounting for 75.4% and 72.9% of isolates, respectively. This finding is consistent with a previous one-year study conducted at the same hospital during 2014–2015, which reported a prevalence of 72.4% for *E. faecalis* [16]. No significant changes were observed in VRE rates or MDR prevalence between the pre-pandemic and in the pandemic/post-pandemic-periods. However, HLGR decreased significantly during the pandemic/post-pandemic period. These temporal findings indicate that enterococcal resistance patterns remained relatively stable across the study periods. Although increased antimicrobial use and potential changes in prescribing practices during the COVID-19 pandemic raised concerns regarding possible effects on antimicrobial resistance patterns [25,26], the observed trends in our cohort cannot be attributed solely to the pandemic, given the influence of other clinical and healthcare-related factors. In addition, the retrospective design and lack of detailed data on antimicrobial exposure, healthcare practices, disease severity, and treatment patterns limit the ability to determine the specific impact of the COVID-19 pandemic on resistance trends. Additionally, coinfections, particularly with *Klebsiella pneumoniae* and *Candida albicans*, were significantly less frequent in the pandemic/post-pandemic era. This reduction may reflect changes in healthcare practices during this period, although causal relationships cannot be established from this retrospective design. During the pandemic, several infection prevention and control measures were reinforced in healthcare facilities, including enhanced hand hygiene compliance, increased use of personal protective equipment, isolation precautions for suspected or confirmed COVID-19 cases, and intensified environmental cleaning and disinfection practices. These measures may have influenced the transmission dynamics of healthcare-associated microorganisms, including enterococci, although their specific impact on enterococcal epidemiology in our setting cannot be determined from the available data.

Several factors were independently associated with mortality among hospitalized patients with enterococcal positive culture, including older age, ICU admission, bloodstream isolation, and the presence of coinfections. Regarding species-specific outcomes, *Enterococcus faecium* was associated with a higher risk of mortality compared with other *Enterococcus* species. Our multivariable analysis demonstrated that *E. faecium* was independently associated with higher in-hospital mortality than *E. faecalis*. However, the mechanisms underlying this association cannot be determined from the present study because antimicrobial resistance profiles, severity of illness, antimicrobial therapy, and other potential mediating factors were not included in the mortality model. Previous studies have reported conflicting findings regarding the contribution of vancomycin resistance to mortality, with some suggesting that resistance itself is not an independent predictor after adjustment for confounding factors [27], whereas others have reported an independent association [28]. Therefore, the higher mortality observed among patients with *E. faecium* in our cohort should be interpreted as an association rather than a direct consequence of antimicrobial resistance. This association may reflect a combination of factors, including differences in patient severity, ICU exposure, infection site, antimicrobial resistance, and treatment-related variables, which could not be fully evaluated in this retrospective study.

In our cohort, VRE prevalence reached 42.9% among *E. faecium* isolates, compared with only 1.6% among *E. faecalis* isolates. This finding is consistent with a local study which identified *E. faecalis* as the predominant species, while *E. faecium* exhibited the highest VRE prevalence and mortality rate [15]. Similarly, international studies have consistently shown that vancomycin resistance is predominantly associated with *E. faecium* rather than *E. faecalis* or other enterococcal species [29,30,31]. This observation is also supported by a recent study from Lebanon, which reported substantially higher rates of vancomycin resistance among *E. faecium* than *E. faecalis* in patients with wound infections, further confirming the predominance of vancomycin-resistant *E. faecium* across different clinical settings and geographic regions [32]. Additionally, four *E. faecium* isolates exhibited concurrent resistance to vancomycin and linezolid. Although uncommon, this phenotype substantially limits therapeutic options and highlights the importance of confirmatory susceptibility testing and individualized infectious disease consultation. Oritavancin has been reported as an off-label therapeutic option in a case of vancomycin- and linezolid-resistant *E. faecium*; however, clinical evidence remains limited, and treatment decisions should be guided by isolate-specific susceptibility results, infection site, and patient-specific factors [33]. Temporal analysis of enterococcal resistance profiles for ampicillin, ciprofloxacin, and vancomycin demonstrated relative stability over the study period, with only minor annual fluctuations. Surveillance of local antimicrobial resistance patterns is essential because changes in resistance profiles directly influence empirical treatment strategies and infection control decisions, particularly in healthcare settings where early appropriate therapy is critical. Similar considerations have been highlighted by Reale et al., who demonstrated the importance of monitoring multidrug-resistant organisms for guiding empirical therapy and infection control interventions [34].

In contrast, HLGR showed a consistent decline over time. Interestingly, the previous one-year study conducted at the same hospital during 2014–2015 reported HLGR rates of 23.7% among *E. faecalis* isolates and 67.9% among *E. faecium* isolates [16]. In comparison, our study demonstrated more moderate HLGR rates in both species, although resistance remained significantly higher in *E. faecium* (32.0%) than in *E. faecalis* (27.1%). These findings indicate a reduction of more than 50% in HLGR among *E. faecium* isolates over time. The observed reduction in HLGR compared with the previous study conducted at the same hospital may reflect several factors, including changes in antimicrobial prescribing practices, antimicrobial stewardship interventions, infection prevention measures, and differences in patient populations over time. However, specific institutional interventions or changes in aminoglycoside utilization during the study period were not evaluated in the present retrospective analysis. Therefore, the observed decline should be interpreted as a temporal epidemiological finding rather than a direct consequence of a specific intervention.

Our study has several limitations. First, it was conducted at a single center, which may limit the generalizability of the findings. Future multicenter studies involving hospitals from different regions of Saudi Arabia are warranted to validate these findings and better characterize national trends in enterococcal epidemiology and antimicrobial resistance. In addition, molecular characterization of the isolates was not performed, which could have provided a better understanding of the epidemiology, transmission dynamics, and potential sources of infection. Moreover, this retrospective study relied on microbiological culture results and routinely collected electronic medical record data. The prevalence of comorbidities may have been underestimated because these were identified from routinely documented clinical records rather than a dedicated disease registry. Therefore, the absence of documentation should not necessarily be interpreted as the true absence of a comorbidity. Furthermore, detailed clinical adjudication of each culture-positive episode was not possible, and differentiation between true infection, colonization, and contamination could not be confirmed in all cases, particularly for urinary and wound specimens. Therefore, the findings should be interpreted as representing hospitalized patients with positive clinical *Enterococcus* cultures rather than confirmed enterococcal infections in every case. In addition, mortality data were obtained from the KKUH electronic medical record system only. Consequently, deaths occurring after discharge may not have been captured, and the reported 30-day mortality may therefore underestimate the true post-discharge mortality. Furthermore, antimicrobial treatment data, including empirical therapy selection, timing of active treatment, and treatment duration, were not available. Therefore, the relationship between resistance patterns, therapeutic management, and clinical outcomes could not be directly assessed.

## 5. Conclusions

This study provides an 11-year overview of *Enterococcus*-positive cultures, antimicrobial resistance patterns, and associated clinical outcomes among hospitalized adults at a tertiary care center. *Enterococcus faecalis* remained the predominant species throughout the study period, while *E. faecium* exhibited substantially higher rates of multidrug resistance and was independently associated with increased in-hospital mortality. Although vancomycin resistance and multidrug resistance remained relatively stable over time, high-level gentamicin resistance showed a significant decline. These findings contribute valuable long-term epidemiological surveillance data that may support local antimicrobial stewardship programs and resistance monitoring. However, because antimicrobial treatment information and detailed clinical severity data were unavailable, the study cannot determine the impact of therapeutic management on patient outcomes. Future multicenter prospective studies incorporating antimicrobial therapy, severity-of-illness measures, and molecular characterization are warranted to further define the relationship between resistance patterns, treatment strategies, and clinical outcomes [17,18,19].

## Figures and Tables

**Figure 1:**
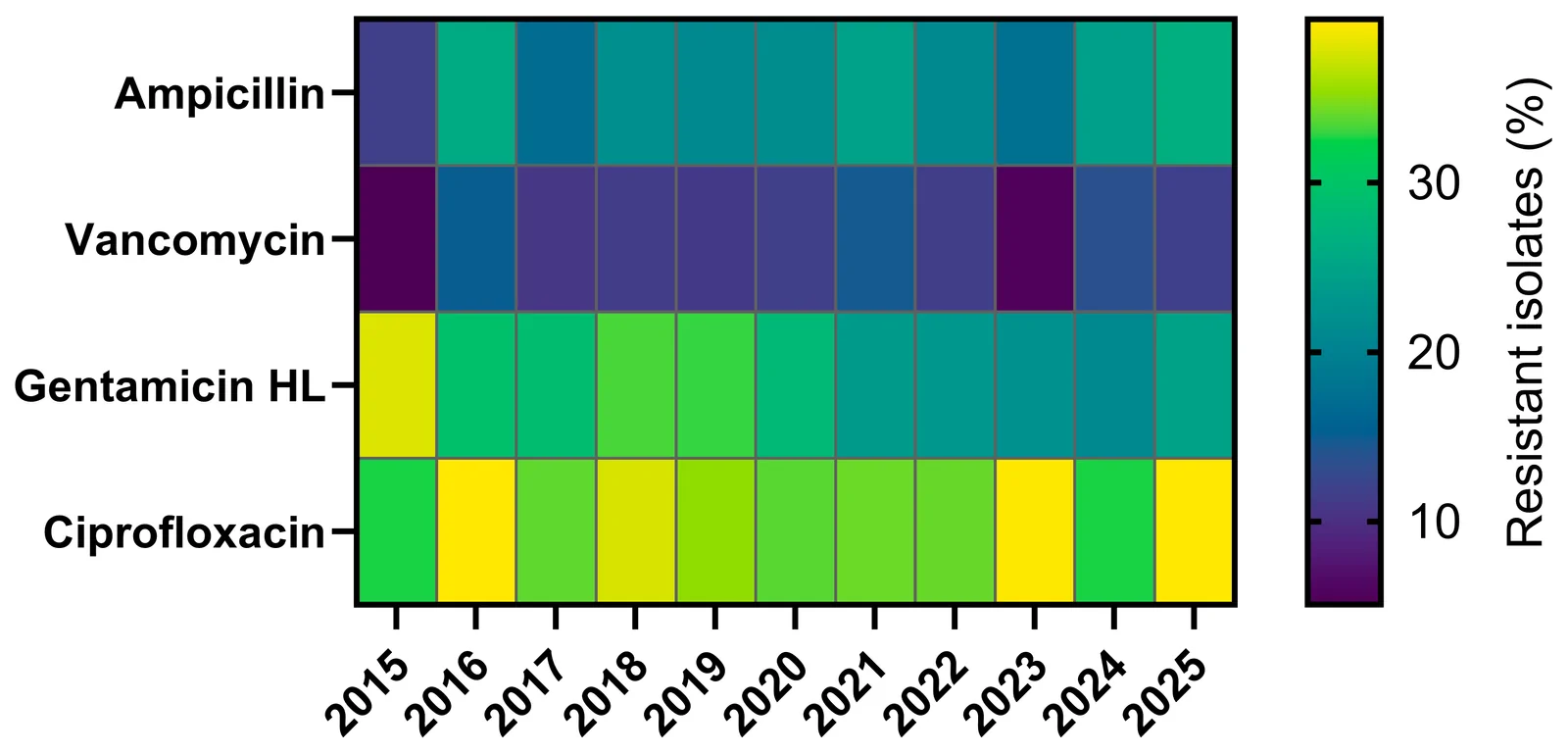
Heat map illustrating temporal trends in the percentage of resistant *Enterococcus* spp. isolates from 2015 to 2025. Resistance rates are shown for ampicillin, ciprofloxacin, HLG, and vancomycin. Percentages were calculated using the number of isolates with an interpretable categorical susceptibility result for each antimicrobial as the annual denominator; isolates with missing or unclassified results were excluded. Annual denominators by year (2015–2025) were: ampicillin, *n* = 95, 186, 232, 295, 227, 217, 232, 203, 217, 223, and 135; ciprofloxacin, *n* = 95, 185, 228, 292, 227, 214, 229, 203, 217, 224, and 134; HLG, *n* = 90, 171, 222, 288, 225, 209, 229, 203, 216, 223, and 133; and vancomycin, *n* = 99, 187, 232, 295, 225, 215, 231, 202, 216, 223, and 136. The annual vancomycin denominators totaled 2261 isolates, with 11 isolates excluded because of missing or unclassified susceptibility results.

**Figure 2:**
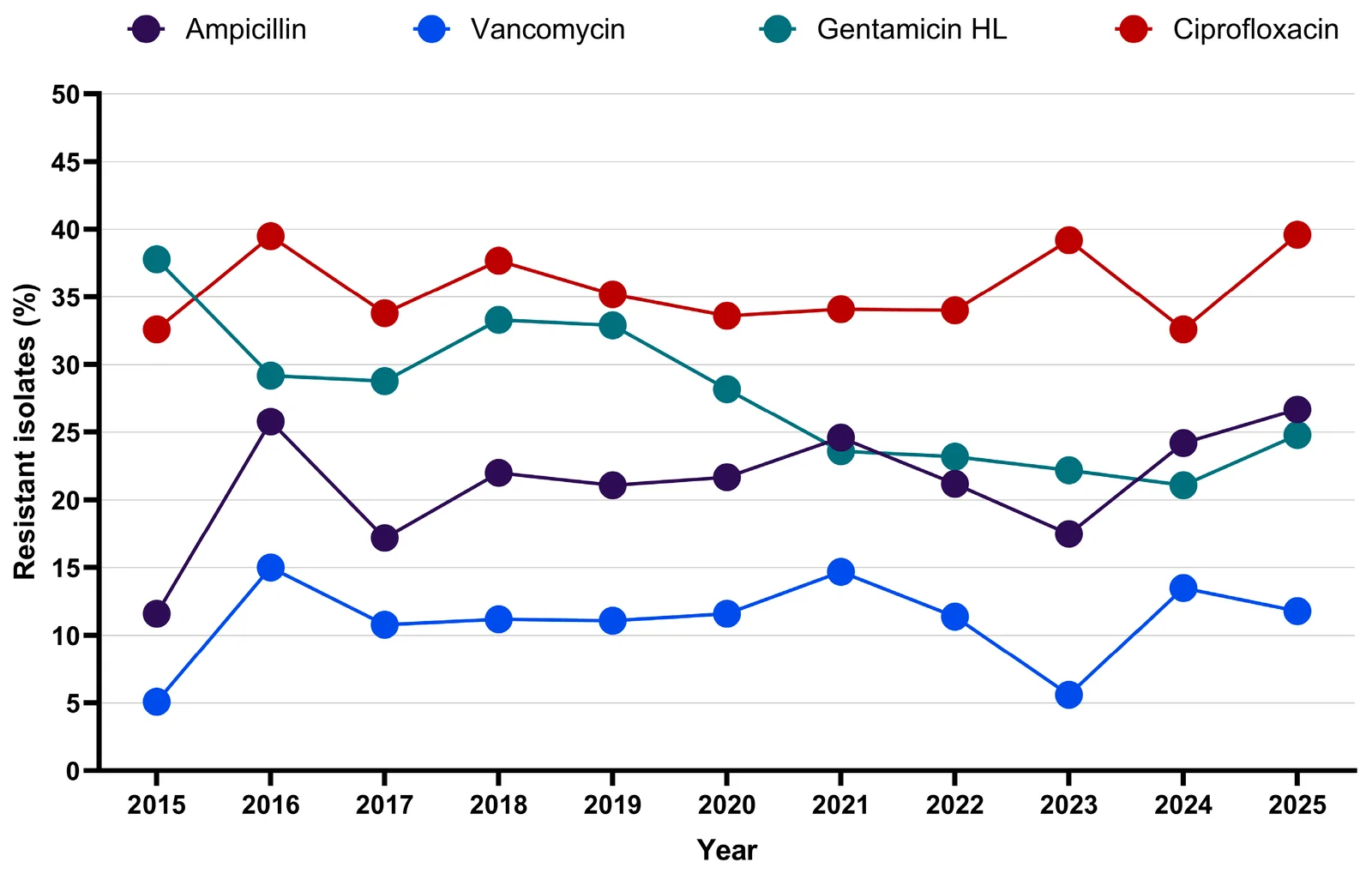
Temporal trends in antimicrobial resistance among *Enterococcus* spp. isolates from 2015 to 2025. The figure shows annual observed percentages of resistant isolates for ampicillin, vancomycin, HLG, and ciprofloxacin. Annual denominators were the numbers of isolates with interpretable categorical susceptibility results for each antimicrobial, as listed in Figure 1 caption for the corresponding year and antimicrobial. Isolates with missing or unclassified susceptibility results were excluded from the calculations.

**Figure 3:**
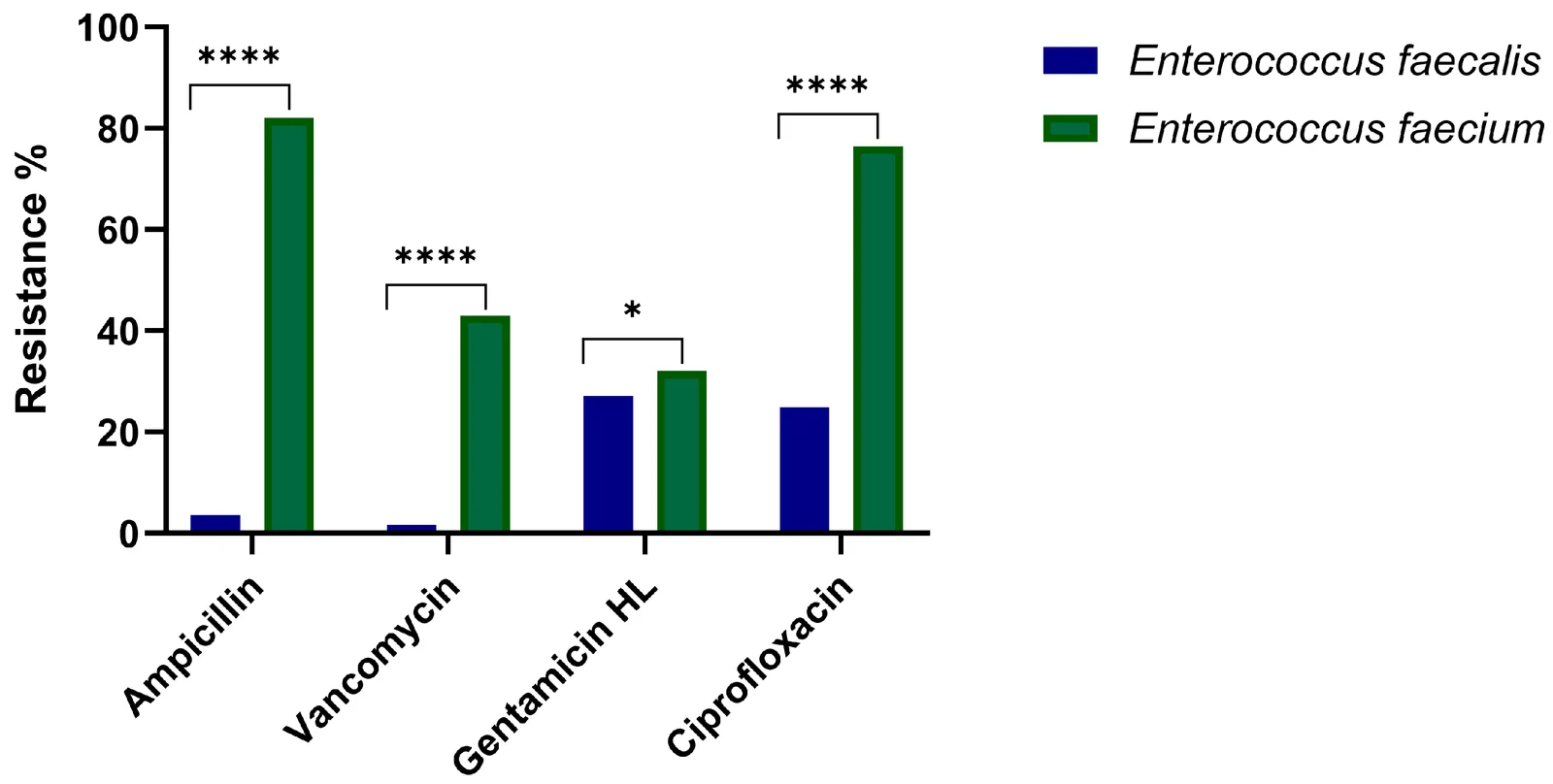
Antimicrobial resistance profiles of *Enterococcus faecalis* and *Enterococcus faecium*. Comparative resistance percentages to selected antibiotics are shown for the two species. Statistical analysis was performed using Fisher’s exact test (two-sided) to assess differences between groups. Significance levels are indicated as *p* < 0.05 (*) and *p* < 0.0001 (****). Denominators are presented as resistant isolates/tested isolates (%): ampicillin, 61/1679 (3.6%) for *E. faecalis* and 386/471 (82.0%) for *E. faecium*; vancomycin, 27/1680 (1.6%) and 201/469 (42.9%); ciprofloxacin, 415/1672 (24.8%) and 360/471 (76.4%); and HLG, 447/1648 (27.1%) and 147/460 (32.0%), respectively. Denominators represent isolates with interpretable susceptibility results for the corresponding antimicrobial.

**Table 1:** Demographic and Clinical Characteristics of Adult Patients with Enterococcal Positive Cultures Pre-pandemic and Pandemic/Post-pandemic period.

Variable	Total (2015–2025) *n* = 2272 *n* (%)	Pre-Pandemic (March 2015–February 2020) *n* = 1085 *n* (%)	Pandemic/Post-Pandemic (March 2020–November 2025) *n* = 1187 *n* (%)	*p* Value
Age, median (IQR), adults ≥18 years only	59 (40–72)	58 (38–72)	60 (41–72)	0.250
Age group (years), adults ≥18 only				
18–39	567 (25.0%)	289 (26.6%)	278 (23.4%)	0.085
40–59	583 (25.7%)	273 (25.2%)	310 (26.1%)	0.637
≥60	1122 (49.4%)	523 (48.2%)	599 (50.5%)	0.301
Sex				
Male	982 (43.2%)	439 (40.5%)	543 (45.7%)	0.013 *
Female	1289 (56.7%)	646 (59.5%)	643 (54.2%)	0.011 *
Unknown/not recorded	1 (0.0%)	0 (0.0%)	1 (0.1%)	
Clinical setting/ward type				
ICU	81 (3.6%)	17 (1.6%)	64 (5.4%)	<0.001 *
Medical ward	116 (5.1%)	16 (1.5%)	100 (8.4%)	<0.001 *
Surgical ward	137 (6.0%)	55 (5.1%)	82 (6.9%)	0.080
Emergency department	1066 (46.9%)	692 (63.8%)	374 (31.5%)	<0.001 *
Oncology/hematology	30 (1.3%)	8 (0.7%)	22 (1.9%)	0.032 *
Other	842 (37.1%)	297 (27.4%)	545 (45.9%)	<0.001 *
Urinary catheter present				
Yes	159 (7.0%)	120 (11.1%)	39 (3.3%)	<0.001 *
No	2113 (93.0%)	965 (88.9%)	1148 (96.7%)	<0.001 *
Catheter type				
Indwelling (Foley/two-way Foley)	131 (5.8%)	97 (8.9%)	34 (2.9%)	<0.001 *
Straight/intermittent	8 (0.4%)	7 (0.6%)	1 (0.1%)	0.032 *
Condom catheter	7 (0.3%)	6 (0.6%)	1 (0.1%)	0.059
Other	13 (0.6%)	10 (0.9%)	3 (0.3%)	0.067
Comorbidities				
Diabetes mellitus	126 (5.5%)	67 (6.2%)	59 (5.0%)	0.246
Hypertension	45 (2.0%)	22 (2.0%)	23 (1.9%)	0.998
Chronic kidney disease (CKD)	11 (0.5%)	1 (0.1%)	10 (0.8%)	0.023 *
End-stage renal disease (on dialysis)	32 (1.4%)	23 (2.1%)	9 (0.8%)	0.010
Cardiovascular disease	58 (2.6%)	29 (2.7%)	29 (2.4%)	0.831
Chronic liver disease	10 (0.4%)	4 (0.4%)	6 (0.5%)	0.861
Chronic lung disease	14 (0.6%)	9 (0.8%)	5 (0.4%)	0.330
Malignancy (solid or hematologic)	135 (5.9%)	59 (5.4%)	76 (6.4%)	0.377
Transplant recipient	1 (0.0%)	0 (0.0%)	1 (0.1%)	1.000
Neurological disease	45 (2.0%)	19 (1.8%)	26 (2.2%)	0.549
Organism				
*Enterococcus faecalis*	1683 (74.1%)	818 (75.4%)	865 (72.9%)	0.187
*Enterococcus faecium*	475 (20.9%)	215 (19.8%)	260 (21.9%)	0.242
Other *Enterococcus* spp.	114 (5.0%)	52 (4.8%)	62 (5.2%)	0.709
Antimicrobial resistance profile				
Vancomycin-resistant Enterococcus (VRE)				
Yes	256 (11.3%)	122 (11.2%)	134 (11.3%)	1.000
No	2005 (88.7%)	959 (88.7%)	1046 (88.6%)	1.000
High-level aminoglycoside resistance (HLAR)				
Yes	918 (40.4%)	482 (44.4%)	436 (36.7%)	<0.001 *
No	1354 (59.6%)	603 (55.6%)	751 (63.3%)	<0.001 *
Multidrug-resistant (MDR)				
Yes	752 (33.1%)	360 (33.2%)	392 (33.0%)	0.973
No	1520 (66.9%)	725 (66.8%)	795 (67.0%)	0.973
**Clinical outcomes**				
Length of hospital stay (median, IQR)	16.9 (5.5–39.0)	16.3 (5.1–40.2)	17.2 (6.0–38.4)	0.470
In-hospital mortality	355 (15.6%)	156 (14.4%)	199 (16.8%)	0.132
30-day mortality	163 (7.2%)	70 (6.5%)	93 (7.8%)	0.232
Coinfection (any)	106 (4.7%)	95 (8.8%)	11 (0.9%)	<0.001 *
*Klebsiella pneumoniae*	90 (4.0%)	81 (7.5%)	9 (0.8%)	<0.001 *
*Candida albicans*	10 (0.4%)	9 (0.8%)	1 (0.1%)	0.018 *
*Candida tropicalis*	4 (0.2%)	3 (0.3%)	1 (0.1%)	0.555

Note: Asterisk indicates *p*-values < 0.05. HLAR: high-level aminoglycoside resistance (high-level gentamicin and/or streptomycin resistance). The prevalence of VRE was calculated using the number of Enterococcus isolates with an interpretable vancomycin susceptibility result as the denominator (*n* = 2261 overall; *n* = 1081 pre-pandemic; *n* = 1180 pandemic/post-pandemic). Isolates with missing or unclassified vancomycin susceptibility results (*n* = 11 overall; *n* = 4 pre-pandemic; *n* = 7 pandemic/post-pandemic) were excluded from the calculations.

**Table 2:** Factors Associated with In-Hospital Mortality Among Patients with Enterococcal Positive Culture.

Variable	Survived *n* (%)	Died *n* (%)	AOR (95% CI)	*p* Value
Age group				
18–39	544 (28.4%)	23 (6.5%)	Reference	
40–59	491 (25.6%)	92 (25.9%)	2.77 (1.60 to 4.98)	0.0002 *
≥60	882 (46.0%)	240 (67.6%)	5.06 (3.08 to 8.72)	<0.0001 *
Urinary catheter present				
Yes	119 (6.2%)	40 (11.3%)	Reference	
No	1798 (93.8%)	315 (88.7%)	1.06 (0.60 to 1.90)	0.83
ICU admission				
No	1865 (97.3%)	177 (49.9%)	Reference	
Yes	52 (2.7%)	178 (50.1%)	28.1 (19.5 to 41.1)	<0.0001 *
Site of isolation				
Urinary tract	1023 (53.4%)	110 (31.0%)	Reference	
Bloodstream	205 (10.7%)	127 (35.8%)	2.69 (1.84 to 3.92)	<0.0001 *
Wound/soft tissue	557 (29.1%)	83 (23.4%)	1.08(0.75 to 1.57)	0.65
Respiratory	4 (0.2%)	2 (0.6%)	1.31(0.11 to 11.0)	0.81
Other	128 (6.7%)	33 (9.3%)	1.60 (0.91 to 2.73)	0.10
Period				
Pre-pandemic	929 (48.5%)	156 (43.9%)	Reference	
Pandemic/post-pandemic	988 (51.5%)	199 (56.1%)	1.18 (0.87 to 1.61)	0.26
Coinfection				
Yes	73 (3.8%)	33 (9.3%)	Reference	
No	1844 (96.2%)	322 (90.7%)	0.50 (0.27 to 0.97)	0.041 *
Organism				
*Enterococcus faecium*	340 (17.7%)	135 (38.0%)	Reference	
*Enterococcus faecalis*	1482 (77.3%)	201 (56.6%)	0.51 (0.37 to 0.70)	<0.0001 *
Other *Enterococcus* spp.	95 (5.0%)	19 (5.4%)	0.62 (0.32 to 1.16)	0.1454

Asterisk indicates *p*-values < 0.05. The outcome was in-hospital mortality (died vs. survived). All variables shown in the table were entered simultaneously into the multivariable logistic regression model. AORs are mutually adjusted for all variables included in the model. Reference categories are indicated as “Reference.”.

**Table 3:** Temporal trend analysis using binomial logistic regression.

Antibiotic	Slope (Log-Odds/Year)	OR per Year	95% CI	*p*-Value
Ampicillin	0.026	1.026	0.991–1.062	0.149
Ciprofloxacin	0	1	0.970–1.030	0.993
Gentamicin HL	−0.069	0.933	0.903–0.964	<0.001
Vancomycin	0.001	1.001	0.957–1.047	0.971

Models were fitted using annual resistant/tested isolate counts with year as a continuous predictor (year − 2015). The OR per year represents the change in odds of resistance for each additional year and was calculated as exp(β_1_). Binomial logistic regression accounted for the number of tested isolates in each year. Year-clustered robust standard errors produced the same statistical inference.

## Data Availability

The data that support the findings of this study are available on request from the corresponding author, E.A.

## Supplementary Materials

The following supporting information can be downloaded at: https://www.mdpi.com/article/10.3390/microorganisms14102175/s1, Table S1: Antimicrobial susceptibility profile of *Enterococcus* spp. isolates, 2015–2025.
