# Supplementary material for: Eleven-Year Trends in Antimicrobial Resistance and Outcomes Among Hospitalized Adults with Positive Enterococcus Cultures
Source: Microorganisms. 2026 Sep 27;14(10):2175. doi: 10.3390/microorganisms14102175 (PMC13707493; doi:10.3390/microorganisms14102175)
Supplement: Supplementary file 1 [file microorganisms-14-02175-s001.zip › microorganisms-4543641-supplementary.pdf]

Table S1. Antimicrobial susceptibility profile of *Enterococcus spp.* isolates, 2015-2025

Antibiotic	Susceptible n/N (%)	Intermediate n/N (%)	Resistant n/N (%)	Total tested, N	*E. faecalis* resistant/tested (%)	*E. faecium* resistant/tested (%)	Other *Enterococcus spp.* resistant/tested (%)
Ampicillin	1775/2262 (78.5%)	0/2262 (0.0%)	487/2262 (21.5%)	2262	61/1679 (3.6%)	386/471 (82.0%)	40/112 (35.7%)
Vancomycin	1999/2261 (88.4%)	6/2261 (0.3%)	256/2261 (11.3%)	2261	27/1680 (1.6%)	201/469 (42.9%)	28/112 (25.0%)
Ciprofloxacin	1281/2248 (57.0%)	166/2248 (7.4%)	801/2248 (35.6%)	2248	415/1672 (24.8%)	360/471 (76.4%)	26/105 (24.8%)
Levofloxacin	1445/2253 (64.1%)	60/2253 (2.7%)	748/2253 (33.2%)	2253	383/1672 (22.9%)	341/473 (72.1%)	24/108 (22.2%)
Linezolid	2189/2232 (98.1%)	33/2232 (1.5%)	10/2232 (0.4%)	2232	6/1655 (0.4%)	4/468 (0.9%)	0/109 (0.0%)
Daptomycin	1604/1673 (95.9%)	55/1673 (3.3%)	14/1673 (0.8%)	1673	0/1280 (0.0%)	14/325 (4.3%)	0/68 (0.0%)
Teicoplanin	1541/1737 (88.7%)	21/1737 (1.2%)	175/1737 (10.1%)	1737	25/1302 (1.9%)	140/364 (38.5%)	10/71 (14.1%)
Tetracycline	745/2256 (33.0%)	21/2256 (0.9%)	1490/2256 (66.0%)	2256	1197/1676 (71.4%)	242/472 (51.3%)	51/108 (47.2%)
Erythromycin	300/2245 (13.4%)	382/2245 (17.0%)	1563/2245 (69.6%)	2245	1072/1666 (64.3%)	427/472 (90.5%)	64/107 (59.8%)
Penicillin	1731/2237 (77.4%)	0/2237 (0.0%)	506/2237 (22.6%)	2237	67/1660 (4.0%)	388/468 (82.9%)	51/109 (46.8%)
Nitrofurantoin	1299/1535 (84.6%)	123/1535 (8.0%)	113/1535 (7.4%)	1535	12/1212 (1.0%)	93/268 (34.7%)	8/55 (14.5%)
High-level gentamicin (synergy)	1603/2209 (72.6%)	0/2209 (0.0%)	606/2209 (27.4%)	2209	447/1648 (27.1%)	147/460 (32.0%)	12/101 (11.9%)
High-level streptomycin (synergy)	1582/2231 (70.9%)	0/2231 (0.0%)	649/2231 (29.1%)	2231	380/1657 (22.9%)	221/470 (47.0%)	48/104 (46.2%)
Quinupristin/Dalfopristin	201/680 (29.6%)	39/680 (5.7%)	440/680 (64.7%)	680	417/444 (93.9%)	8/193 (4.1%)	15/43 (34.9%)
Tigecycline	690/693 (99.6%)	0/693 (0.0%)	3/693 (0.4%)	693	0/447 (0.0%)	3/204 (1.5%)	0/42 (0.0%)

Note. Percentages use the number of isolates tested for the specific antimicrobial as the denominator. Intermediate includes I and SDD results. Other *Enterococcus spp.* includes all species other than *E. faecalis* and *E. faecium*.
